# Comparing the Effect of Animal-Rearing Education in Japan with Conventional Animal-Assisted Education

**DOI:** 10.3389/fvets.2017.00085

**Published:** 2017-06-07

**Authors:** Yuka Nakajima

**Affiliations:** ^1^Department of the Modern Social Studies, Otemae University, Nishinomiya, Hyogo, Japan

**Keywords:** animal-rearing education, school animals, animal-assisted education, Japanese educational system, intellectual and emotional development

## Abstract

An increasing number of teachers are introducing animals into their class so that pupils foster cognitive, physiological, and social skills through their interaction with animals. Along with such an educational style termed animal-assisted education (AAE), Japanese formal education has also utilized animals for education. Japanese animal-rearing education is unique regarding the following two points: (1) it takes the form of “education through assisting animals” rather than “animals assisting education” and (2) animal rearing is embedded in formal education. While conventional AAE expects the benefit from the social support of animals, Japanese animal-rearing education expects benefit from nurturing and caring for animals. The present study aims to identify effective methods for using animals for education by highlighting the benefits of Japanese animal-rearing education. An overview of Japanese animal-rearing education is followed by a critical review of empirical studies of conventional AAE and Japanese animal-rearing education. Despite the differences in the educational styles, it was found that both systems commonly help children adapt to school. Additionally, conventional AAE were effective in enhancing cognitive and athletic ability of students and foster social skills, while Japanese animal-rearing education enhanced academic knowledge and skills and cultivated sympathy for animals and other people. Furthermore, it was demonstrated that the experience of raising animals affects children’s development for a long time even after children stop raising animals. In order to determine the effect of animal presence at school, however, more empirical studies with various viewpoints are necessary for both styles of education. Concerning Japanese animal-rearing education, the effects of the differences such as the amount of exposure to animals, developmental stage or character of individual children, the types of animals need to be controlled for a more sophisticated examination. Empirical studies show that preadolescence is one of the periods in which animal rearing has the greatest impact on children’s development. It is suggested that through the program of raising school animals, conventional AAE obtains more a variety of effects in their interaction with animals.

## Introduction

Since 1980s, accumulating data has indicated the possibility that children’s relationship with companion animals has a therapeutic [e.g., Ref. (1, 2)] as well as psychological [e.g., Ref. (3, 4)] and behavioral [e.g., Ref. (5, 6)] impact on children’s development. Such findings have encouraged teachers to introduce animals into their class so that pupils foster cognitive, physiological, and social skills through interacting with animals (7, 8). The number of teachers who bring animals into their class has been increasing (9, 10).

### Conventional Animal-Assisted Education (AAE)

The conventional style of utilizing animals for education, termed AAE, is used in many European countries. One of the most popular AAE programs is the “school dog” where teachers regularly (1–5 days/week) take their dogs into the classroom as school dogs. Beetz (9) reported that more than 500 teachers in these countries work with their school dogs. The goal of a teacher–school dog team is to influence social behavior, socioemotional competence, and the empathy of the children and to improve the classroom environment, motivation, and discipline (9, 11, 12).

The canine-assisted reading program is also a popular AAE program. The goal of the program is to support children with poor reading skills and who are reluctant to read aloud (10). This program is widely popular among elementary schools and at libraries of more than 42 states in the United States (10) as well as in Canada, Australia, the United Kingdom, Italy, and India (13). Reading Education Assistance Dogs (READ) is one of the first programs that was established, in 1999, and it was followed by many other canine-assisted reading programs, including the All Ears Reading and Share program, Literacy Education Assistance Pups (LEAP), Paws to Read (10).

International guidelines for AAE have been adopted (14, 15), which suggests that education with the help of animals has become more prevalent worldwide. Systematic guidelines for introducing animals into the classroom have been developed (16, 17). The necessity and importance of education assisted by animals have been acknowledged in the society.

In such a context, the possibility and the impact of school dogs are also being investigated in Japan (18). The introduction of school dog is, however, rather an unusual style of AAE in Japan. Japan has developed a unique style of AAE for keeping animals in schools as a part of formal education for more than 100 years.

### Animal Rearing Embedded in Japanese Formal Education

Traditionally, but not compulsorily, Japanese kindergartens and elementary schools have had animals such as rabbits and chickens in school for education. Japanese formal education started in the late 19th century. Matsuda described how animals were introduced into formal education, explaining the kinds of animals that are appropriate to keep at a school, how to rear them, and the effective usage and impact of having animals as teaching tools (1908, 1911, and 1913). In his science-teaching plan, Matsuda (19) used chickens kept at school as a teaching tool to help pupils learn chickens’ form, habits, and biology. These papers by Matsuda show that interaction with and the rearing of animals has been employed as a method of teaching in Japan. Rabbits and chickens were recommended to keep because of their gentle nature and because they are easy to care for and tame (20, 21), and so are they found in schools in today.

The Japanese program is unique regarding the following two points:

1. Japanese animal-rearing education takes the form of “education through assisting animals” rather than “animals assisting education.”
2. Animal rearing is embedded in formal education.

First, “rearing of animals” is centered on the method of AAE in Japan. That is, while “children getting assisted by animals” is a popular approach in other nations and areas, Japanese schools try to foster children’s knowledge, health, and emotional intelligence through their “assisting” animals. Regarding this point, raising school animals is the means and also the goal of education. As the second point, the Japanese national government formally promotes the “rearing of school animals” as an educational tool. National guidelines for keeping animals in school also exist. According to this policy, animals are raised by pupils as an educational tool from preschool through junior high school. Schools also make an annual academic schedule that includes animal rearing, and children raise animals as an academic activity.

### The Goals of the Present Study

The goal of the present study is to quest a new possibility of AAE through focusing on Japanese animal-rearing education.

When we think about the bond between humans and pets, its relationship is reciprocal; pets provide us social support, and we also support pets. The presence of pets alleviates stress (22, 23), improves psychological well-being (24, 25), and reduces physiobiological risks [e.g., Ref. (26, 27)]. The presence of pet also physically and psychologically supports children. Children receiving inpatient psychiatric treatment show improvement in their state of mind after therapy incorporating a dog (1). Reviewing articles on animal-assisted intervention for autism spectrum disorder, O’Haire (2) suggested that autistic children become relaxed and ease their stress through interaction with animals, which helps them lessen their aggressive emotions.

Meanwhile, as the other side of the relationship with pets, we are also benefitted through raising and caring for pets. Children develop nurturance skills through the rearing of pets (5, 28). Intimate relationship with pets also fosters empathy for others (3) or prosocial attitude (4) among children.

Thinking through this reciprocal viewpoint, conventional AAE is benefitted mainly from the supportive nature of animals. On the other hand, Japanese animal-rearing education reflects the other side—the benefit through caring for animals.

Contrasting these two educational systems—conventional AAE and Japanese animal-rearing education, the present study is to examine the merits of Japanese AAE and analyze the factors that make Japanese AAE effective and its problems. Highlighting on a Japanese animal-rearing education, the present study proposes another possibility of education through animal presence that may complement conventional AAE.

### The Course of the Present Study

First, this study presents an overview of Japanese animal-rearing education. Then, comparing the conventional AAE and Japanese animal-rearing education, the present study examines their similarities and differences. It also examines the factors that make Japanese animal-rearing education effective through the reports from school teachers and veterinarians. Finally, the issues that need to be solved for future study are discussed.

## Japanese Animal-Rearing Education—Its Overview

Rearing school animals is incorporated into formal education in Japan, with school animals employed as teaching tools. In the course of study established by the Ministry of Education, Culture, Sports, Science and Technology (MEXT), there are guidelines for keeping and utilizing school animals for each grade and covering several subjects. The following excerpt of the course of study (29) indicates how the rearing of school animals is embedded in school education.

### Living Environment Studies for First and Second Grades

In the subject living environment studies, animal rearing is included as an academic activity for first- and second-grade pupils. One of the goals of living environment studies is to “help pupils become interested in the relationship between themselves and people around them, society and nature through concrete activities and experiences (29).”

According to this goal, the course of study for living environment studies poses the necessity of raising animals and growing plants so that pupils become interested in the habitat of animals and plants and their changes and growth. The course of study also suggests that raising animals and growing plants help pupils realize that these are living and growing entities, become familiar with living things, and cherish them. In order to meet this goal, the course of study for living environment studies recommends that schools have children keep animals for two successive years, from first through second grade. Although the course of study does not specify the kind of animals, Shimano (30), a school inspector for MEXT, discusses that the species of animals to be familiar to pupils and are robust, resistant to disease, and suitable for school and the surrounding environment. Generally, keeping guinea pigs or hamsters in the classroom is popular, and teachers then encourage the children to take care of the animals.

### Period for Integrated Studies for Higher Grades

Older children in the third through the sixth grade learn about raising animals and having respect for their life through the course, the period for integrated studies.

In Japanese education, the period for integrated studies is unique on the point that it does not have a concrete, uniform style of learning. The goal and the contents of the period for integrated studies are entrusted to each school. Schools are just required to conduct cross-synthetic learning activities in the period for integrated studies, such as international understanding, information, environment, welfare/health based on pupils’ interests and concerns.

In order to reach this goal, many schools utilize school animals as learning tools, because learning respect for life is taken as one of the most important tasks for children to achieve in elementary school. As a school inspector for MEXT, Shimano (31) discusses that through raising animals children learn and synthesize knowledge and experience concerning “respect for life.” They learn about life in various subjects—domestic science, living environment studies, Japanese, science, and so on. Such knowledge they gain from those subjects, however, is just a partial knowledge of life. Shimano (31) maintained that the actual experience of raising, caring for, and having contact with animals synthesizes this partial knowledge of life, and, further, lets children learn the importance of life—not only for animals but also for friends and other people.

**Figure 1:**
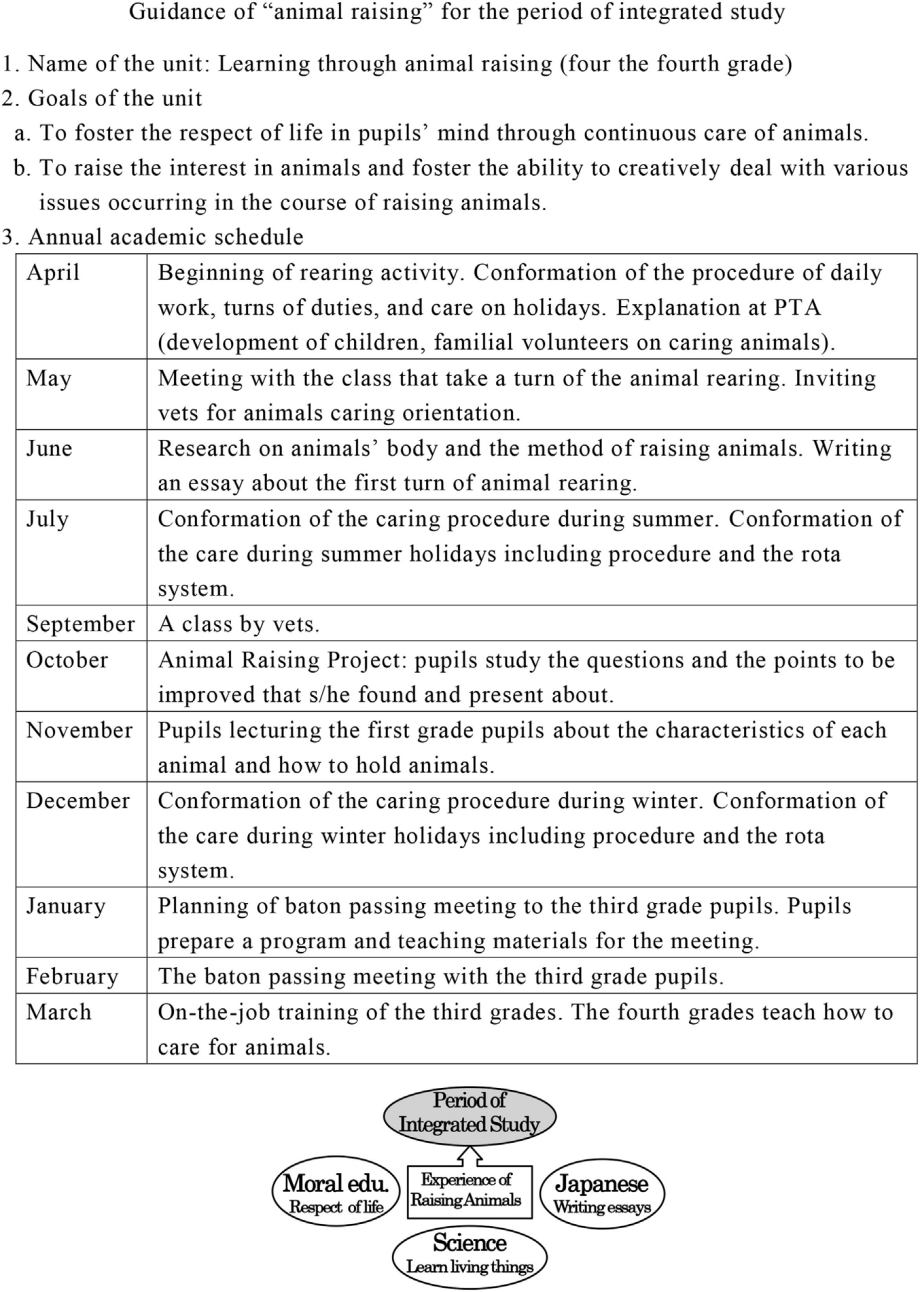
Annual educational plan of PIS using animal rearing as an educational tool. Source: data adapted from Ref. (32).

In the context of the period for integrated studies, schools formulate an annual teaching program. Schools set minute goals (Figure 1) for the academic year. On the basis of these goals and manuals, the activities for each month were determined. Animal rearing can also be related to other subjects, including Japanese, science, music, art, or moral education so that children can attain the required goals in each area.

### Moral Education and Science

The course of study for moral education, from the first grade to the sixth grade, underscore that children should cherish animals and plants and respect the life of other people, animals, and themselves. The background to this idea is that today children have scarce opportunities to cherish and respect lives. Nakamura (33) investigated children’s ideas about life among 372 fourth and fifth graders and found that, when asked whether a deceased person would revive, 33.9% answered, “yes” and 31.5% answered, “I don’t know.” In such a climate of children’s thought for life, the Central Council for Education strongly proposed moral education based on actual experience as a way of helping children think about life and death. Thus, the course of study for moral education attaches importance to the rearing of animals and growing plants, which can lead children to experience the importance of life and to value nature, animals, and plants (34).

The course of study for science also considers observation and interaction with familiar animals as an important teaching practice. Also, the course of study for science explains that, through caring for animals, children learn about the mechanisms of animals’ bodies and the differences and commonalities between animal bodies and the human body. The course of study expects that raising animals, including fish and insects, at school can cultivate a scientific outlook in children.

Interestingly, the course of study emphasizes the linkage between science and moral education. Hioki (35), a senior specialist for curriculum in MEXT, explained that the ability to show respect for life or to have a protective attitude toward animals is cultivated through touching, interacting with, and raising them for a certain period of time. Thus, the course of study presents learning about the physiobiological aspects of animals as a way of guiding children to cultivate consideration for them and other living things.

### Special Activity

Another popular way of rearing school animals is to join the animal-rearing committee that constitutes the student-body activity. The course of study requires that children participate in student-body so that they build good social relations and develop a self-motivated attitude. The student committee, including the animal-rearing committee, usually consists of children from upper grades. The members of the committee take care of the animals on behalf of the other pupils and report the current condition of and problems related to raising the animals to the student-body activity (36).

### The Network of the Support

MEXT also makes an effort to enlighten teachers about preferable animal-rearing practices in schools. Commissioning a study with a private research institute, MEXT distributed a handbook, *Desirable Animal Rearing at* School (37) to kindergartens, elementary schools. The handbook covers a broad range of topics: the relationship between children’s psychological/physical development and animal rearing; desirable ways of caring for animals both in terms of the animals’ welfare and child development; issues and countermeasures related to animal rearing at school; and examples of practical instruction so that each school can refer to it when owning and caring for animals.

One more unique aspect of raising animals in schools in Japan is the assistance that the veterinary community provides. MEXT requires schools to consult with veterinarians about proper animal rearing (29, 38). Thus, prefectural veterinary associations support schoolteachers throughout the country. Veterinarians provide medical care for school animals, advise teachers on how to raise animals, and help children cultivate a humane attitude toward animals. Thus, veterinarians are also strong supporters of the practice of animal rearing in schools.

### Animals in Japanese Elementary Schools—General Situation

Then, how and what kinds of animals are kept in schools? Where in the school are the animals located? The current study presents the status of the practice of raising school animals according to a study by Hatogai (39). Hatogai, in 2003, randomly chose 866 elementary schools out of all the elementary schools in Japan and sent them a questionnaire about animals reared in schools (mammals and birds). Out of the 866 elementary schools, a total of 579 responded to the questionnaire.

Out of the 579 schools, 88% owned mammals or birds. Many schools kept multiple types of animals. The most popular animal was the rabbit (78.7%), followed by chickens (65.5%), small birds (17.0%), duck (4.3%), hamster (3.6%), and guinea pig (2.0%).

**Figure 2:**
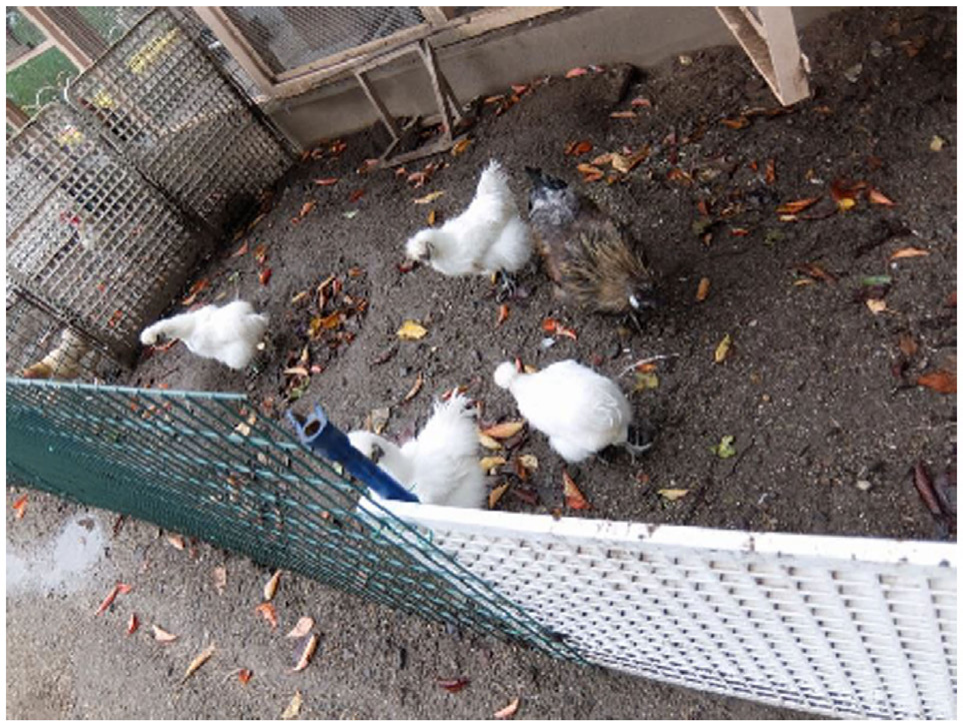
Animals in the animal house of the schoolyard.

**Figure 3:**
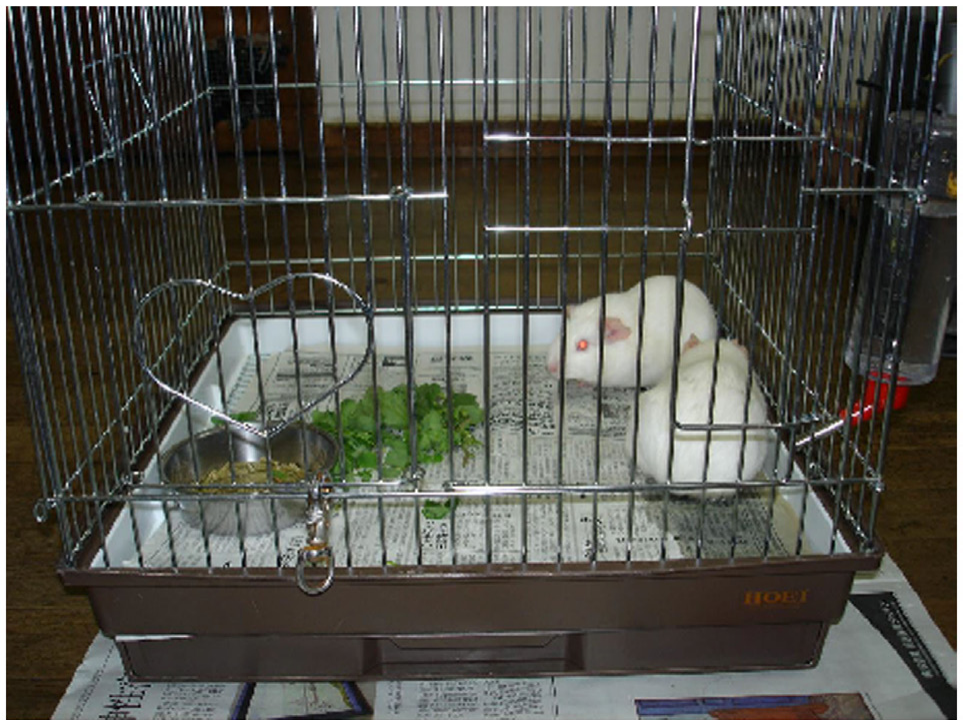
Animals in a cage in the classroom.

The number of most of these animals was single to five (63.55% of rabbits, 62.2% of chickens, 95.5% of ducks, 94.1% of hamsters, and 100% of guinea pigs). Almost all rabbits (97.7%) and chickens (98.8%) were kept in an animal house located in the schoolyard (see Figure 2), while hamsters (94.1%) and guinea pigs (66.7%) were kept inside the school building, including in hallways and classrooms (see Figure 3). MEXT, supported from the Japan Veterinary Association, has presented guidelines for the habitat of each kind of animal, including rabbits and chickens. The goal of this detailed advice is to keep animals clean and hygienic and ensure their welfare in accordance with Act on Welfare and Management of Animals (1973, amended in 2012) (40) as well as help children learn respect for life, experience pleasure in caring animals, and find that they can easily care for animals.

The daily work involved in rearing animals includes feeding them and cleaning their living space. In most schools, children mainly engage with caring for animals whether or not the activity is an academic subject (living environment studies or the period for integrated studies) or a committee project. Preparation of food for the animals is also included in the work of rearing at some schools. Children care for the animals before the start of the school day, during lunchtime, or after school. Children at some schools enjoy playing with the animals after taking care of them, while others just spend time tending them.

Among the schools that own animals, more than 70% responded they utilize school animals for teaching living environment studies. In the event of an animal’s death, almost 80% of schools replied that they would bury the animal with the pupils taking part (39). Teachers think that raising animals fosters respect for life, kindness to animals, a sense of responsibility, and consideration for others in children. The background of teachers’ such belief is that children take care of animals on their own. In line with the school curriculum, teachers observe the pupils’ activity, and those in upper grades help the ones in lower grades develop their skills of caring for animals. The background of such animal-rearing education is the belief that taking care of animals helps children’s scientific and psychological development.

Hatogai (39) found that, at more than 70% of schools, the pupils on the animal-rearing committee and teachers in charge of the committee took care of the animals. At approximately 20% of the schools, all pupils in a particular grade and their teachers were in charge of caring for the animals (for living environment studies or the period for integrated studies). If that is the case, then is it mainly members of the animal-rearing committee who develop respect for life, kindness toward animals, responsibility, and consideration for others? Tanaka and Tachikawa (41) conducted a study with 330 elementary schools and found that more than 70% of pupils were reported to visit the school animals during their free time. They also reported that pupils at schools in which they can easily access the animals visited them significantly more often as compared to pupils at schools in which access to the animals is somewhat more difficult. Moreover, pupils visited animals significantly more frequently when schools make arrangements for the space or time to see the animals as compared to schools that do not. Although pupils, other than those on the animal-rearing committee, may only occasionally, and not sufficiently, engage with school animals, through living environment studies or through play, they also cherish the animals, which may lead to their developing consideration for animals as well as respect for life.

The nationwide survey on animal rearing at school has not been conducted for more than a decade since Hatogai (39). The proportion of schools that keep animals, the number and types of animals, the place these animals are located could have changed since then. A new survey is required to clarify how the situations surrounding animal rearing have changed.

## The Effect of Animal-Rearing Education—Comparison with Conventional AAE

Then, what kind of impact does animal-rearing education have and how do the effects differ from the one through conventional AAE? To make these points accurate, I rely on the studies that specifically cite animal utilizing education at school as their main focus.

Since both the studies of animal-assisted and animal-rearing education are rather new, accumulation of empirical study of the education is not abundant. In such a situation, the first principle of inclusion was the publication of the original research in a peer-reviewed scientific journal. Mainly Educational Resource Information Center (ERIC), Teacher Reference Center, Psychology and Behavioral Science Collection, MEDLINE, and J-STAGE were used to search articles. Search terms were “animal-assisted education,” “school,” “animal,” and “pet.” The presentation abstract of academic conferences were also included. Further, the three criteria were as follows: (1) the articles in which subjects were older than university students were excluded because the review focused on the impact on children. (2) The study of which context was not school situation was excluded even when subjects are children. (3) The articles that targeted to assess the quality of the animals for AAE is also excluded. The reviewed articles concerning conventional AAE are summarized in Table 1, while the reviewed articles concerning Japanese animal-rearing education are summarized in Table 2.

**Table 1:** Original studies included in the review of conventional animal-assisted education.

Reference	Study	Animal	Presence of the animal	Population/age group	*N*	Significant effect
Beetz et al. (42)	The dog as a stress modulator for children with insecure attachment	Dog	During experiment	Male children (age 7–11 years) with insecure attachment, having learning, emotional, and behavior disorders	47	Physiological stress response got lower and relaxed with the dog’s support compared with other conditions

Beetz (9)	Socioemotional experiences emotion-regulation strategies	Dog	1 day/week during the experiment (one of three dogs)	Third-graders (age 8–9 years)	46	Positive attitude toward school and positive emotions concerning learning

Gee et al. (43)	Athletic performance tasks in the presence or absence of dog	Dog	During experiment	4–6-year-old preschooler (five typical and nine developmentally delayed)	14	In dog presence faster completion of the task and increased performance accuracy

Gee et al. (44)	Following instruction in motor skill tasks in the presence of dog, stuffed dog, or human	Dog	Chances to get familiar with dogs prior to the experiment	4–6-year-old preschooler (five typical and nine developmentally delayed)	11	Better adherence to instructions when a dog was a model performer

Gee et al. (45)	Adherence to instructions in the presence of dog, stuffed dog, or human	Dog	Multiple visits prior to the experiment	3–5-year-old preschool children (five typical and seven developmentally delayed)	12	Fewest prompts needed in real dog condition, most prompts in the human condition

Gee et al. (46)	Cognitive tasks in the presence of dog, stuffed dog, or human	Dog	Twice-weekly visits prior to the experiment	3–5-year-old preschool children (seven typical and five developmentally delayed)	12	Fewer irrelevant choices in the real dog condition

Hergovich et al. (47)	Before and after survey of the effect of presence of a dog	Dog	Every day during the experiment (one of three dogs)	First-graders (most of them were immigrants)	46	More empathy to the animal, more field independence, more social integration

Kotrschal and Ortbauer (11)	Before and after survey of observation of children in classroom	Dog	1 month, every day (one of three dogs)	Children (mean age: 6.7 years)	24	More social integration, less aggression, and hyperactivity, more attention toward teacher

Tissen et al. (12)	Three times before and after survey of social training with dogs, without dogs, and dog attendance (no social training).	Dog	90 min/week over 10 weeks	Third-grade children	230	Reduced the frequency of being the victims of open as well as relational aggression

**Table 2:** Original studies included in the review of Japanese animal-rearing education.

Reference	Study	Animal	Location and purpose	Program	Population/age group	*N*	Significant effect
Fujisaki (48)	Videotaping three age group children’s animal rearing	Rabbits, guinea pigs	Kindergarten	Preschool education	Preschoolers (age 5–6, 4–5, and 3–4 years)	60/53/20	Older children had more biological knowledge about rabbits and personalized the rabbits more than younger children

Gunma Veterinary Medical Association (49)	Repeated survey on age different groups			Six-month rearing animals	First-graders and fifth-graders	22/26	Increase of sympathy toward others among first-graders, increase of prosocial attitude among fifth-graders

Gunma Veterinary Medical Association (50)	Repeated survey on keeping condition and age, different groups		Classroom, animal house in the school site	1 year rearing animals	Fourth-graders, fifth-graders, and sixth-graders	114/116/114	No significant difference among rearing conditions for sympathy for others and prosocial behavior

Iwama et al. (51)	The relationship between past experience of nature and present view on life				University students	411	The experience of animal rearing contribute to developing views on animal life

Nakagawa and Muto (52)	Differences of written essays between the two animal-rearing approaches	Rabbits, chickens, and goat	Animal house in the school site	Period for integrated study, committee activity	Fourth- through sixth-graders	191	Higher composition skills and socioemotional intelligence in study-based group compared to committee-based group

Nakajima et al. (53)	Repeated survey comparing animal rearing group and non-rearing group, animal rearing at school and at home	Rabbits, chickens	Animal house in the school site	Period for integrated study	Fourth-graders	768	Appropriate rearing leads to increase/less decrease of knowledge of animals, school adaptation, sympathy for animals, warmth toward people, and prosocial attitude

Maruyama et al. (54)	Before and after survey of experiment and control group, age difference	Guinea pigs	Classroom	11-month rearing animals	Second- and third-graders, fourth-and fifth-graders	443/410	Older experiment group improved empathy for school animals compared to control group

### Conventional AAE

#### Effect on Athletic Performance

Gee et al. (43) investigated whether the presence of a dog affects the performance of motor skills tasks. They found that children completed the tasks faster when the dog was present than when the dog was absent. Gee et al. (43) suggest that the presence of the dog made the children feel more relaxed, less stressed, and more motivated, which lead to an improved speed in task completion. The presence of the dog, however, did not lead to a better performance in tasks.

#### Effect on Adherence to Instructions

Gee et al. (44) examined if the presence of a real dog relate preschoolers’ ability to follow instruction in motor skill tasks. Children performed the tasks in four conditions; the presence of a real dog, the presence of a stuffed dog, the presence of a human, and performing alone. It was found that children tend to adhere to instructions better when the dog was acting as a model compared to other conditions. Gee et al. (44) suggest that a dog could act as a model of good behavior in different situations. They infer that children might be motivated to perform the tasks in the same way the dog did, which encouraged children to adhere to instructions. This indicates that it is because of the unpredictable nature of the dog, relative to a human performer, made the level of tandem tasks difficult. However, when children performed the tasks in tandem with the dog, the adherence to the instructions was poorer than when they performed with a human or stuffed dog.

Gee et al. (45) examined the effect of the dog’s presence on instruction adherence during cognitive tasks such as object/picture recognition. They investigated if the number of instructional prompts such as “face forward” or “pick one of the object/picture” differs according to the difference of conditions: a dog’s presence, a human’s presence, and a stuff dog’s presence. They found that the dog’s presence condition required fewer instructional prompts relative to the other two conditions. They argue that the result of their experiments deny the common assumption that the presence of a dog can be distracting for children during the execution of cognitive tasks. Gee et al. (45) infer that children enjoyed the cognitive tasks more with the attached dog than with the stuffed dog or the human, which motivated children for the task. However, if the degree of attachment was the key, there is a question whether the three conditions were truly homogeneous.

#### Effect on Cognitive Performance

Gee et al. (46) examined the effect of the dog’s presence on 12 children’s performance of cognitive tasks such as choosing a picture of an object that match another under three conditions: a dog’s presence, a human’s presence, and a stuff dog’s presence. They found that the presence of the dog resulted in fewer erroneous choices than other conditions. They suggest that the presence of the dog reduce children’s stress and make them more relaxed, which helped children to focus their attention on the demands of the tasks.

#### Effect on Improving Social Behavior

Kotrschal and Ortbauer (11) investigated the impact of dog’s presence in classroom on children’s social behavior. After 1-month control period in the absence of dogs, one of the three dogs was present in the classroom during the 1-month experimental period. Twenty-four children’s behavior in the classroom during both periods were videotaped and analyzed. It was found that social cohesion of the class increased when the dog was present when compared to that of the control period; behavioral extremes such as aggressiveness and hyperactivity decreased, withdrawn individuals became socially more integrated, and children paid more attention to the teacher. They suggest that children’s contact with and interest in the dog lead to children’s behavioral changes. But, the mechanisms that the dog’s presence increased children’s behavior are not made clear.

Tissen et al. (12) examined the effects of social training with/without a dog on social behavior, empathy, and aggression in 230 children. They employed three experimental conditions for the 10-week program; social training without dogs, social training with dogs, and dog attendance but no social training. Variables were assessed by teachers before the start of, after the completion of, and 3 weeks after the program. They found that, after program, children under the social training with dogs significantly reduced the frequency of being the victims of open as well as relational aggression. Social behavior and empathy were also improved over time, but there were no significant difference among program. This result suggests that, just the presence of the dog might have equivalent effect for children’s social behavior, empathy, or aggression with social training. But, more elegant research design that includes the group of children in the condition “no social training program, no contact with dogs” should be necessary.

#### Effect on Relaxation after Stressful Tasks

Beetz et al. (42) examined the dog’s possibility as a stress modulator. They hypothesized that children with insecure attachment can profit more from social support by a dog compared to a friendly human or a toy dog during stressful task. In the experiment, 47 insecurely attached male children with learning and emotional and behavior disorder underwent Trier Social Stress Test for Children in which children are asked to make a short presentation in front of a committee of unfamiliar adults and perform a mathematical task. Social supporters, including the dog, were present during and 30 min after the stress test. It was found that the physiological stress response of children supported by a dog get lower after the test compared to other conditions.

#### Effect on Positive Attitude toward School

Beetz (9) investigated the effect of a school dog–teacher-team on depression, emotion regulation, and social and emotional school experiences of third graders. The data of intervention class (with a dog presence) and control class (with no dog) were collected twice; before the dog was introduced and 2 weeks before the school year. It was found that the 1 day/week presence of a school dog showed stronger improvement in a positive attitude toward school and positive emotions concerning learning compared to a control class over the course of a year. However, more study is needed as to whether these positive effects are due to the presence of the dog or due to other factors, including the relationship between school dog and the teacher.

#### Effect on Cognitive and Social Development

Hergovich et al. (47) examined the effect of the presence of a dog in classroom on children’s cognitive and social development. Of 46 participants, 43 of them were immigrants. Experiment class (with a dog presence everyday) and control class (with no dog) were surveyed twice; at the beginning of the dog presence and 3 months later. It was found that children in the experimental class showed enhancement of field independence and empathy with animals in comparison with the control class children. Further, after the 3-month presence of a dog, children in experiment class showed better integration into the class group, compared to the beginning of the dog presence.

However, more study is needed to elucidate especially the relationship between enhanced field independence and the presence of a dog.

#### Effect on Reading Proficiency

Although empirical study was not obtained, it is reported that the presence of dogs help children with reading. Reading proficiency improves through reading practice (55). Poor readers, however, tend to be reluctant to read aloud, which can lead to a vicious cycle (56). In a canine-assisted reading program, children read aloud to well-trained supportive dogs, which enable children to relax and enjoy improving their reading performance (13). It has been reported that the program increased confidence, a feeling of comfort, and motivation for reading among children (57).

Due to the supportive environment that dogs provide, they are often brought into special educational classes to help children with disabilities. Anderson and Olson (58), for example, investigated the effect of AAT with a dog present in a class for six children with emotional disorders. Through qualitative analysis, the authors reported that the dog’s presence improved the children’s overall emotional stability and their attitudes toward school and their lessons.

### Japanese Animal-Rearing Education

#### Effect on Intellectual Development

Fujisaki (48) investigated 133 preschoolers (60 5–6-year-old children; 53 4–5-year-old children, and 20, 3–4-year-old children) who raised rabbits at a kindergarten concerning their biological knowledge and understanding of rabbits’ psychology. Fujisaki found that the 5–6-year-old children spent a significantly longer time with the rabbits, spent a longer time cleaning the rabbits’ habitat, and communicated with the rabbits more frequently than did the younger children. The 5–6-year-old children also had significantly more biological knowledge about rabbits and personalized the rabbits more than did the younger children. Among the 5–6-year-old children, those who spent a longer time with the rabbits had significantly more biological knowledge about rabbits and personalized the rabbits more than did those who spent less time with the rabbits. Paradoxically the amount of biological knowledge is proportional to the amount of personalization. The personalization is considered a naïve belief characteristic to early childhood. However, analyzing the communication of university students and children’s parents with rabbits, Fujisaki (48) found that these adults showed a significantly higher personalization in their communication than children, which suggests that children’s personalization of rabbits might not be the manifestation of naive belief. It proves, rather, that children, based on rabbits’ behavior, infer that rabbits have the same emotions as people and that rabbits are social living beings just like human beings.

These results might be partially brought by the effect of intellectual development of children. However, the participating children had reared animals continuously since they entered this kindergarten. It can be thought that such a rich experience to rear and interact with animals fostered the development of the knowledge about animals and the theory of mind. However, comparison with no rearing group should be necessary to examine the true effect of animal rearing.

#### Effect on Composition Skills and Socioemotional Intelligence

Nakagawa and Muto (52) investigated whether differences in the animal-rearing approaches influence children’s intellectual development. They compared two approaches to animal rearing in a school: committee-based animal rearing and animal rearing as the period for integrated study. While study-based animal rearing is included as a regular educational activity, committee-based rearing does not have a concrete educational goal or a syllabus. Nakagawa and Muto (52) hypothesized that educational goals should be one of the pivotal factors for children’s intellectual as well as emotional development in raising animals. They evaluated such children’s development through their essays about animal rearing. They analyzed 191 essays submitted for an essay competition concerning the raising of school animals; 94 essays written by children engaged in study-based animal rearing (study group) and 97 essays by children engaged in committee-based animal rearing (committee group).

The criteria for evaluating the essays were composition elements and emotional expression. It was found that the study group wrote more words as compared to the committee group. The study group also showed higher composition ability and higher emotional expression compared to the committee group even when the number of words was controlled.

Basically, the daily responsibilities of children caring for animals did not differ between the period for integrated studies group and the committee group. They both fed the animals, gave them water, and cleaned their habitat. The data showed, however, concerning emotional expression, that the study group’s scores were higher in terms of expressing animals’ emotions and of insight into those emotions, in terms of sympathy and nurturance for animals, and in terms of identifying with friends and teachers who were caring for animals together.

Then, what created such difference between study group and committee group? Nakagawa and Muto (52) suggested two points. First is children’s deep involvement with animals. In the period for integrated studies group, children have ample opportunity to play and interact with animals, which enabled children to take an interest in animals and feel intimacy with and attachment to each animal. Nakagawa and Muto (52) hold that such experiences in the period for integrated study help children foster an attachment to animals, which was embodied as significant emotional expression in the essays.

The second was the utilization of raising animals as an educational opportunity. Nakagawa and Muto (52) reiterated the importance of teachers’ educational concerns in raising animals. In the case of period for integrated study, schools create a yearly syllabus for raising animals. Along with this syllabus, each of the raising activities is utilized with educational meanings. Children are, for example, to interact with animals so that the activity helps them become interested in animals, observe them, and feel an attachment to them.

Nakagawa and Muto (52) hold that such an educational involvement enables children to foster attachment and sympathy toward others. However, it should be taken into account that the background including school or their developmental stages, the type of animals they reared, the way of rearing animals are all different in the research of Nakagawa and Muto (52).

#### Effect on Empathy for School Animals

Maruyama et al. (54) investigated the effects of classroom pets on Japanese children’s empathy for school animals and other people. The participants were 853 pupils (in grades two through five) from nine elementary schools. Experiment groups were given two to three guinea pigs to keep in class for 11 months. The control group did not engage in raising animals. Children’s empathic attitudes toward animals and humans were measured at the beginning and end of the 11-month period. Pupils were further divided to create two groups by grade. For younger children (second and third graders), their scores (pre- vs. post-test score) showed no significant improvement in empathy toward animals or humans. Among older children (fourth and fifth graders), those who reared guinea pigs showed significantly greater score improvement (pre- vs. post-test score) in empathy for animals as compared to the control-group children. The results of Maruyama et al. (54) indicate that the effect of rearing animals is useful in cultivating empathy for animals in older children.

#### Effect on Sympathy, Prosocial Attitude, and School Adaptation

Nakajima et al. (53) conducted a repeated cross-sectional study with 768 fourth-grade elementary school children, from 12 schools. The study consisted of a questionnaire that was conducted: (i) prior to the intervention (Time 1; T1), (ii) at the end of the 1-year intervention (Time 2; T2), and (iii) 1 year following the conclusion of animal-rearing intervention (Time 3; T3). Five variables (knowledge of animals, sympathy for animals, warmth toward people, prosocial attitude, and school adaptation) were measured to investigate the impact of animal raising at school on children’s psychological development. Because the outcome variables of the three groups at baseline were not comparable, Nakajima et al. (53) subtracted T1 value from T2 value or T3 value of each variable and used the variation of the five variables in order to examine the psychological development of the children.

In the investigation, importance was attached to the following two points. First is the quality of animal rearing carried out by the children. Visiting the animal houses at several schools, the authors found that the quality of care for the animals differed by school. Some schools did not clean the animal house sufficiently; some schools did not care for the animals well during holidays or vacation; or some schools did not consult veterinarians, which resulted in a poor living environment and the death of the animals. As Vidovic et al. (4) reported attachment to animals influences children’s psychological development including consideration for others. Nakajima et al. (53) predicted that differences in the quality of animal care influences children’s consideration for animals, which also affects differences in their consideration for their friends or other people. Children were classified into three groups according to the quality of animal rearing: appropriate-rearing group (247 children at four schools), inappropriate-rearing group (203 children at three schools), and non-rearing group, i.e., the control group (318 children at five schools), which did not rear school animals (see Table 3).

**Table 3:** Evaluation of the animal rearing [source: data adapted permission from Ref. (53)].

Group of the school (*n*)	Appropriate rearing (247)	Inappropriate rearing (203)
Average of the group’s points	M	M
1. Involvement of pupils (total)	3.00	−2.00
1.1. Feeding	(1.00)	(0)
1.2. Care on holidays	(1.00)	(−1.00)^a^
1.3. Interaction with animals	(1.00)	(−1.00)^b^
2. Involvement of school (total)	1.75	−1.33
2.1. Degree of interest	(0.75)	(−0.33)
2.2. The box in the animal houses	(1.00)	(−1.00)^c^
3. Educational plans	1.00	−1.00
4. Education on animals’ death	0.50	−0.67
5. Support from vets (total)	2.00	0.67
5.1. Introductory lecture	(1.00)	0.33
5.2. Visiting and support	(1.00)	0.33
6. Health condition of animals^d^	1.00	−1.00
Average of the total evaluation	9.25	−5.33

*^a^According to the records of vets and narrative from teachers in charge, only security guards took care of animals on holidays*.

*^b^Pupils in some schools wore plastic gloves and masks when they took care of animals despite the advice of vets that such gears are unnecessary because bird flu outbreak was already over. Moreover, a school prohibited pupils not to visit the animal house other than cleaning the house because it was located at dangerous place*.

*^c^To protect animals against heat and cold, vets require schools to put shelter box in the animal house in the case the house is located outside the building*.

*^d^According to the records of vets, the health condition of the animals in these schools was poor*.

After a 1-year period in which animals were raised, it was found that appropriate-rearing group, compared to the control group, showed a lower decrease in school adaptation during T1 through T2. However, inappropriate-rearing group, as compared to appropriate-rearing group, showed a higher decrease in all five variables during T1 through T2. During T1 through T3, 1 year after the end of animal rearing, as compared to the control group, appropriate-rearing group showed a lower decrease in all variables except for knowledge of animals. However, inappropriate-rearing group showed the highest decrease in prosocial attitude in the three groups. It is reported that warmth to other people decreases as the grade in school advances and students approach puberty (59). The control group of Nakajima et al. (53) also showed a decrease of warmth toward others or prosocial attitude. It was found that animal rearing at school has the impact of diminishing this decrease when children raise animals appropriately.

The second point is whether children keep animals at home. Pets are considered as family members [e.g., Ref. (60, 61)]. Having contact with pets at home should have a considerable influence on children’s psychological development, including consideration for others (3, 4). The ownership of pets at home—outside of rearing animals at school—might also influence children’s psychological development.

In order to examine the effect of keeping pets at home, Nakajima et al. (53) divided the three groups into six groups according to whether or not the child keeps pets at home.

It was found that, after 1 year of animal rearing, those who appropriately reared school animals but had no pets at home showed a lower decrease in the sympathy for animals and prosocial attitude compared to those who inappropriately reared school animals regardless of having pets at home. During T1 through T3, 1 year after the end of animal rearing, again, those who appropriately reared school animals but having no pet at home showed a lower decrease in sympathy for animals, school adaptation, and prosocial attitude as compared to those who were in control group and kept pets at home.

Nakajima et al. (53) clarified the impact of rearing animals at school on children’s psychological development. In particular, Nakajima et al. (53) demonstrated that caring for animals at school may have an identical impact on children’s psychological development with keeping pets at home. Moreover, Nakajima et al. (53) showed that, in contrast, inappropriate animal rearing might cause children to be insensitive toward having a prosocial attitude or sympathy for animals.

Nakajima et al. (53) should be one of the largest investigations on the impact of the school animal to children’s psychological development. However, the design of the study makes it unclear if the conditions of the groups are comparable. There is, for example, a possibility that general learning environment or socioeconomic factor of each school is different. Also, the individual pupil’s attitude toward animals, including the attachment to animals or the like and dislike of caring animals, should critically affect the sympathy for animals and other variables. Thus, not just examining the differences of schools, more sophisticated grouping such as the degree of attachment or attitude of caring of each child should be necessary for a more thorough and elegant study.

#### Replication of Nakajima et al. (53)

Gunma Veterinary Medical Association (49) partially replicated Nakajima et al. (53). In order to investigate the impact of animal rearing on prosocial attitude and sympathy for others, 22 first graders and 26 fifth graders participated in the study before and 6 months after starting animal rearing. They found that sympathy toward others among the first graders rose after animal-rearing activity. Meanwhile, prosocial attitude of the fifth graders also rose after the animal rearing.

Another research of Gunma Veterinary Medical Association (50) made a comparison on the effect of the difference of rearing conditions after one-year of animal rearing. The data were collected before and 1 year after starting the animal rearing. The rearing conditions were rearing in classroom, rearing in animal house located in school site, and control group (no rearing activity). Data were analyzed according to each grade. However, for each grader, no significant difference among the rearing condition was found concerning prosocial activity and sympathy for others.

#### Long-lasting Impact of Rearing Animals

Furthermore, it has been found that such experiences in rearing and caring for school animals have an impact beyond childhood (51). Iwama et al. (51) investigated 411 university and graduate students concerning the relationship between nature experiences in childhood and their view of life. The authors found that experiences with animals, including raising school animals or keeping pets in childhood, had a significant relation to students’ sense of life. Through the experience of rearing school animals or pets, students developed a realization of the significance of life by witnessing the birth of animals or seeing them rear their offspring.

### Common Effects between Two Educational Systems

As long as these empirical studies show, dogs are overwhelmingly popular in conventional AAE, while dogs are rarely kept in Japanese animal-rearing education. Also, in conventional education, dogs usually do not live in school. They are teachers’ or handlers’ pets and visit school. In order to take enough rest, most dogs do not go to school every day or multiple dogs go to school by turn (9, 47). Also, in most cases, teachers take care of the dogs. On the other hand, in case of Japanese animal-rearing education, animals live in the animal houses set in the site of the school. Children take care of animals, and teachers follow children. Teachers take an initiative on making an educational plan and work together with veterinarians.

Despite such environmental differences, animals in both educational systems commonly have the effect to help children adapt to school including school adaptation (53), enhancing positive attitude toward school and learning (9), social integration (11, 47), and a reduction in the stress response (42). The result of Nakajima et al. (53) suggests the presence of animals in the “here and now” is crucial. They reported that the school adaptation of children caring for animals at school shows a lesser decrease when compared to the control group who did not care for animals at school. Also, children who properly cared for animals at school show less decrease of school adaptation compared to those who do not care for animals at school but keep pets at home. These findings suggest that even if children keep pets at home, the presence of animals at school is pivotal for adapting to school.

Another underlying reason for animals’ impact on school adaptation would be that children feel animals do not evaluate them. Dogs, for example, provide a safe, playful, and caring learning environment (13). Because we feel that animals do not assess or despise us, we feel less stress when we are with animals compared to that of human friends (23). This sense of security helps children adjust to school environment and motivates them to learn.

### Difference of Both Animal Educations

Other than school adaptation, the effect of conventional AAE can be classified into two categories. The first is enhancement of cognitive and athletic ability. This includes enhancement of field independence (47), fewer irrelevant choices in cognitive tasks (46), enhancement of attention (44, 45), and faster and more accurate completion of the athletic task (43). The second is an enhancement in the social skill to get along with others at school, which includes less aggression and hyperactivity (11), more attention toward teacher (11), reduced victims of open as well as relational aggression (12).

Meanwhile, Japanese rearing animals has effect on two aspects other than school adaptation. The first is the enhancement of academic knowledge or skills including biological knowledge of animals (48, 52), theory of mind (48), composition skills (52). The second is the consideration for animals and other people, including sympathy or empathy for animals (53, 54), socioemotional intelligence (52), warmth or sympathy toward others, and prosocial attitude (49, 53). This leads to the respect of life.

Concerning the development of individual ability, conventional AAE has impact on the development of fundamental cognitive or physical ability that support academic learning. On the other hand, Japanese animal-rearing education is effective for enhancing academic achievement. One of the reasons for such a difference would be the difference of the educational goals. Especially, Japanese education expects that the experience of animal rearing lead to the acquisition of knowledge and skills in other subjects. The results of empirical studies show that such a goal of Japanese animal-rearing education is, though partially, attained.

Concerning the effects on the social relationship, conventional AAE emphasize on the change in an individual’s behavior, such as reducing aggression and hyperactivity. Meanwhile, Japanese animal-rearing education rather focuses on psychological development such as sympathy and warmth for others or prosocial attitude.

There would be two reasons as to why Japanese animal-rearing education showed effect on the development of such a tender mind. One reason is the similarity of experience in keeping animals at school and pets at home. It can be thought that since rearing animals at school is a similar experience to that of keeping and caring for pets at home, the same impact that pets have on child development was also found. Empirical studies show that the relationship with animals foster prosocial mind among children (3, 4). Investigating the parents of 701 preschoolers and elementary school children, Fogel and Melson (5) found that interaction with animals, especially for boys, would be a highly effective way for cultivating tenderness toward others. The high school students were asked why keeping animals is good for their development in the investigation by Robin et al. (62); to this, more than a half answered that it is because they learn responsibility through keeping. Nakajima (63) suggested that through rearing, caring for, and playing with school animals, children infer what makes animals happy and what makes them frightened. By inferring what is in an animal’s mind, children cultivate consideration for others.

Another possible reason would be the cultural difference in terms of the sense of value. Markus and Kitayama (64) argue that western individualistic society, including United States, encourage people to be unique, express themselves, and promote their personal goals, while collectivistic society, including Japan, encourage people to adjust themselves to the group to which they belong, to infer what runs in one another’s minds, and to be sympathetic toward others. Such a difference in the construal of self—independent and interdependent—was found, for example, to affect mothers’ developmental expectation toward their children (65, 66). While American mothers highly expected their children to acquire social skill including taking leadership, being assertive, or getting along with friends, Japanese mothers especially expected their children to be obedient to others or not to bother others. It is suggested that such a social climate in Japan was reflected in the educational goal of rearing animals in each school, which foster sympathy for others or prosocial attitude in children.

## Actual Effort of Schools to Make School Animal Rearing Effective

How then does rearing animals in school contributes to the development of children’s mind? Analyzing the differences in appropriate-rearing and inappropriate-rearing groups, Nakajima et al. (53) suggested four factors that are pivotal to differentiating the effect of rearing school animals. Reports from school teachers and veterinarians depict their actual effort and ingenuity for these four factors.

### Clear Goal of the Study and Well-Thought-Out Learning Program for the Goal

It was found that the appropriate animal-rearing education often embeds animal rearing in the academic goals of the school (53). Maruyama et al. (67) and Saito et al. (32) reported their school’s plan for a period for integrated studies class in which all children to have ample opportunity to be involved for a 1-year period in animal rearing during their time in elementary school. Suzuya Saitama Municipal Elementary School (68) researched how children view animals before it wrote the guidelines. Based on the results of this research, teachers defined the structure, theme, and goals of the class for each month. Also, schools often utilize animal rearing by linking it with other subjects. Takahashi (69) showed that children learn the biology of an animal in science class and the importance of life in Japanese and in moral education class before taking the period for integrated studies class. With such prior knowledge, the experience of having contact with and raising an animal in the period for integrated studies class encourages children to realize what actual animals are like and the importance of life.

### Dedicated Involvement and Ingenuity of Teachers

Teachers’ support and guidance are also essential for children’s development through animal rearing. Fujii (70) reported that she made a handbook so that the children could write their findings, including physiobiological information, behavior, and the needs of the animals they were caring for. She also encouraged the children to write what they needed to do or what they could do for the animal. She found that the handbook helped the children summarize their scientific knowledge and reflect on their thoughts about this activity. Teachers also support children to develop autonomous and problem-based thinking. Saito et al. (32) as well as Mitsuhashi (71) depict the “taking-over meeting” that fourth graders hold with third graders at the end of the academic year. At the meeting, fourth graders teach third graders physiobiological and behavioral information about each animal. Annually, the fourth graders create materials to introduce each animal, tell the lower grades how to prepare food and clean the animal house, and make a presentation. They teach the third graders how to hold the animals, the DOs and DON’Ts of animal rearing, and invite the third graders for 1 month of on-the-job training in animal rearing.

### Learning from the Death/Sickness of Animals

Another difference between the appropriate-rearing group and inappropriate-rearing group was the lessons on the death of school animals. It is obvious that the response of the adults around the children regarding the sickness or death of animals has a significant impact on the children’s psychological development. Yarrow et al. (72) reported that children learn to have concern for others through observing adults’ behavior or emotion toward others. Ascione (73) also argued that children emulate adults’ actions of violence or cruelty toward others. Nakajima et al. (53) suggested that adults’ cruelty or apathy regarding animals’ suffering or death should mar children’s sympathy for animals.

Teachers report that holding a memorial ceremony for deceased animals, including writing letters or saying goodbye to them, soothes the sorrow of children, helps them realize the gravity of life, and aids them in understanding that the deceased will never come back [e.g., Ref. (67, 69, 71)]. Morishita (74) showed that when “Leo-kun,” a guinea pig raised in the class for living environment studies, died, children discussed about the death of Leo-kun during moral class, which helped them see the rearing of animals from the animals’ perspective. Saito et al. (32) also reported that death and followed memorial ceremony of “Yellow,” a chicken, changed children to read books or research on a computer to find out what they could do for animals or what was the best way to raise animals. Ishijima (75) also reported children’s psychological development through taking care of a disabled rabbit. Experiencing the death/sickness of animals, children gain a deeper insight into life by themselves and learn to cooperate with their friend to autonomously cope with the difficulties.

### Support from Veterinarians, Families

Support from veterinarians is also an indispensable factor. Vets visit a school to give a class on the animal’s biology and how to raise the animal (32, 76), examine the animals, and occasionally give support and advice to teachers (77). Some local vet associations produce a handbook (76) or a wall newspaper (78) on how to care for school animals so that children find rearing enjoyable. Families of children also help animal rearing during vacations by keeping the animals at their home (71) or by coming to the school with their children to take care of the animals (32).

## Limitation of Research in Japanese Animal-Rearing Education

It was found that Japanese animal-rearing education affects children’s academic performance as well as prosocial attitude. However, for more sophisticated examination in the future, the next four points need improvement.

First, it is still unclear who benefits from raising animals at school. Some children care for the animals and some just say hello or goodbye to the animals when they see animals while arriving at or leaving school. Some pupils care for the animals eagerly and properly and some do not. The frequency of contact and intimacy with animals may therefore be different among these various groups. The effects of these differences on the amount of exposure to animals on children’s development need to be investigated. Also, children’s difference other than animal rearing such as character or scholastic aptitude need to be included into examination in order to demonstrate the impact of animal rearing on children’s psychological development more accurately.

The second point is that there are developmental differences among children. Some pupils rear animals as part of living environment studies in first or second grade, some do so as the period for integrated studies study in the third or fourth grade, and some do so as an animal-rearing committee activity in fifth or sixth grade. Such differences in developmental stages should be made clear.

The third point has to do with the difference in types of animals. Some schools have reported on pupils caring for fish; some on small animals kept in the school building, including the classroom; and some on chickens or rabbits kept in an animal house located outside the school building. The method of rearing or the degree of intimacy with animals may be different when the species of animals are different.

The fourth point is the criteria for measuring the quality of animal rearing. Nakajima et al. (53) showed that inappropriate animal rearing can have an undesirable effect on children’s socioemotional development. They evaluated “inappropriateness” from the viewpoints of veterinarians’ observations and educational aspects. The definition of inappropriateness is, however, still unclear and arbitrary.

## Conclusion

The goal of the present study was to examine a new possibility of AAE through focusing on Japanese animal-rearing education and its effect.

The empirical studies revealed that the effect of conventional AAE was mainly seen in terms of the enhancement of cognitive and athletic abilities as well as that of social skills at school, such as less aggression and hyperactivity or paying more attention to the teacher. On the other hand, the effect of Japanese animal-rearing education was seen in the enhancement of academic knowledge or skills as well as consideration for others, including sympathy for animals and others or prosocial attitude. Furthermore, it was demonstrated that the experience of raising animals affects children’s development for a long time even after children stop raising animals.

Such a difference in effects stems from the different relationship with animals between these two educational systems—getting assisted by animals versus assisting and caring for animals. Adopting animal-rearing education, popular in Japan, would provide a new possibility for conventional AAE. Preadolescence is one of the periods in which animal rearing has the greatest impact on children’s development (28, 54). Erikson (79) proposed that the elementary school years occur in the stage of industry, during which children enhance their potential through challenging and accomplishing tasks. It is suggested that through the program of raising school animals, conventional AAE obtain more variety of effects in interaction with animals.

In order to determine the effect of animal presence at school, more empirical studies with various viewpoints and sophisticated design are needed for both of conventional AAE and Japanese animal-rearing education. Attachment to animals, for example, is one of the pivotal factors that differentiate the impact of the bond between humans and animals (3, 4). Nakajima (80) reiterated the necessity to differentiate between rearing and attachment. Attachment and rearing are predicted to have a strong correlation. Even so, the relationship and functional difference between attachment and rearing should be investigated.

Further, the research on the balance between cost and effectiveness of using animals for education is necessary. Hatogai (39) reported that one of the most prevalent disadvantages found among schoolteachers concerning the caring for animals in school is the care that must be provided during holidays. Zasloff et al. (81), analyzing data from 37 teachers in 35 elementary schools, also found this same disadvantage of keeping animals in the classroom. Further, Matsuda (82) wrote more than 100 years ago about the difficulty of caring for animals during holidays. These data suggest that the disadvantages of caring for animals, especially providing care during holidays and vacation, have been historical as well as intercultural problem that must be coped with.

Nakajima (80) asks whether “easy-to-care” rearing truly has less value in terms of children’s development. She suggests that intellectual or socioemotional development is not the only or the absolute index of the merit of raising school animals. She proposed that just keeping and watching goldfish or crawfish should have some desirable impact if children are attached to them. Of course, animals should be cared for in close adherence to Act on Welfare and Management of Animals (1973, amended in 2012) (40). Based on animal welfare, the best balance between “easy rearing” and “beneficial rearing” should be investigated.

## Author Contributions

The author confirms being the sole contributor of this work and approved it for publication.

## Conflict of Interest Statement

The author declares that the research was conducted in the absence of any commercial or financial relationships that could be construed as a potential conflict of interest.
